# Relationship of Hearing Loss to Parkinson’s Disease, Dementia, and APOE Genotype in Adults

**DOI:** 10.3390/medicina60050703

**Published:** 2024-04-25

**Authors:** Chih-Hung Cha, Tsu-Kung Lin, Ching-Nung Wu, Chao-Hui Yang, Yi-Wen Huang, Chung-Feng Hwang

**Affiliations:** 1 Department of Otolaryngology, Kaohsiung Chang Gung Memorial Hospital, Chang Gung University College of Medicine, No. 123, Dapi Rd., Kaohsiung 83301, Taiwan; d070025@cgmh.org.tw (C.-H.C.);; 2 Department of Neurology, Kaohsiung Chang Gung Memorial Hospital, Chang Gung University College of Medicine, Kaohsiung 83301, Taiwan; 3 Center of Parkinson’s Disease, Kaohsiung Chang Gung Memorial Hospital, Kaohsiung 83301, Taiwan

**Keywords:** hearing loss, dementia, Parkinson’s disease, apolipoprotein E

## Abstract

*Background:* Hearing loss has been recognized as a risk factor for dementia and non-motor features of Parkinson’s disease (PD). The apolipoprotein E (APOE) protein contributes to maintenance and repair of neuronal cell membranes, causing age-related disorders. This study aimed to analyze the impact of hearing loss on cognitive impairment, PD severity, and APOE gene expression in these patients. *Methods:* A total of 72 out-patients diagnosed with either PD or hearing loss were enrolled in this study. The hearing assessment included pure-tone audiometry, speech reception thresholds, and speech discrimination ability. Dementia was assessed by filling out the Clinical Dementia Rating and Mini-Mental State Examination questionnaires. The severity of PD was assessed using the Modified Hoehn and Yahr scale. Blood samples were tested for the gene expression of APOE. *Results:* Out of the 72 cases, there were 44 males and 28 females, with an average age of 64.4 ± 9.1 years. A total of 41 out of 72 cases had dementia and had a worse hearing threshold than those without dementia (47.1 ± 24.4 vs. 31.7 ± 22.1 dB, *p* = 0.006). A total of 58 patients were diagnosed with PD, with 14 of them classified as having severe symptoms (Modified Hoehn and Yahr scale > 2). Patients with severe PD were found to have a worse hearing threshold (49.6 ± 28.3 vs. 30.3 ± 17.8 dB, *p* = 0.028) and higher prevalence of dementia (12/14 vs. 18/44, *p* = 0.006). Among 10 individuals with the APOE ε4 gene, the prevalence of dementia was higher than those without the ε4 allele (9/10 vs. 32/62, *p* = 0.036). *Conclusions:* Hearing loss is common in severe PD and in dementia patients. Severe PD has a negative impact on the hearing threshold and cognitive dysfunction. Patients with APOE ε4 have a higher prevalence of dementia.

## 1. Introduction

Around 466 million people worldwide have disabling hearing loss, affecting approximately one third of people over 65 years of age [1]. Hearing loss has a negative impact on various aspects of a human’s life, including social isolation, emotional stress, pessimism, amnesia, learning disability, inconvenience, etc. [2]. Hearing loss may impair cognitive function in different ways: increased cognitive load, change in brain structure, and reduced social engagement [3]. Recently, hearing loss has been thought to be linked with cognitive decline and recognized as a risk factor for neurodegenerative diseases, such as dementia and Parkinson’s disease (PD) [3,4,5,6]. 

Currently, more than 55 million people have dementia worldwide. Every year, there are nearly 10 million new cases [3]. Dementia results from a variety of diseases and injuries that affect the brain. Alzheimer disease is the most common form of dementia and contributes to 60–70% of cases. Other types include vascular dementia, dementia with Lewy bodies (abnormal deposits of protein inside nerve cells), and frontotemporal dementia (degeneration of the frontal lobe of the brain).

PD primarily affects the elderly, with a prevalence of more than 1% in the population above 70 years old. Age is the most important independent risk factor of PD [7,8]. The severity of PD is classified into seven stages, according to the Modified Hoehn and Yahr scale. Younger-age patients tend to have slighter symptoms and earlier stages of PD. Typical symptoms of PD are hand resting tremor, muscle stiffness, bradykinesia, and gait disturbance. Apart from the decline in motor and cognitive functions, it is gaining attention that most PD patients have age-related hearing loss (ARHL, or presbycusis), which is a common non-motor feature of PD [5]. Untreated hearing loss is likely to worsen cognitive dysfunction and to lead to PD dementia [9,10]. However, previous studies lacked information about the severity of hearing loss, dementia, and PD. 

The gene encoding apolipoprotein E (APOE) is located on chromosome 19. APOE contains four exons and can translate into a lipoprotein of 299 amino acids, regulating metabolism of lipids [11,12]. This lipoprotein is crucial to maintain and repair neuronal cell membranes, and some of its genetic variants are the strongest genetic risk factors contributing to age-related degenerative disorders, such as dementia, generalized atherosclerosis, and age-related macular degeneration. 

Individuals carrying the APOE ε4 allele are at risk of developing Alzheimer’s diseases (AD) as well as other forms of dementia [13,14]. There are six allelic combinations of the gene encoding APOE: ε2/ε2, ε2/ε3, ε2/ε4, ε3/ε3, ε3/ε4, and ε4/ε4. Individuals carrying the APOE ε4 allele have increased risk of developing AD as well as other forms of dementia, including PD dementia and Lewy body dementia [13,14]. Emerging evidence indicates that the APOE genotype may influence the PD course, although clinical and neurochemical correlates have not been completely established. APOE ε4 alleles were more commonly found in PD patients characterized by prominent amyloidopathy and non-motor symptoms. The presence of ε4 leads to more widespread Lewy body brain accumulation, higher amyloid-β-plaque deposition, and α-synuclein pathology. PD patients carrying APOE ε4 were found to have faster cognitive decline and were at higher risk of progression to dementia [14]. 

This study aimed to analyze the impact of hearing loss on cognitive impairment, PD severity, and APOE gene expression in these patients.

## 2. Materials and Methods

### 2.1. Patient Recruitment

We randomly recruited participants diagnosed with either ARHL or PD at our out-patient clinic, including from the otolaryngology and neurology departments. Initial assessment included pure-tone audiometry (PTA), speech reception thresholds, and speech discrimination ability. Relevant dementia questionnaires were filled out, including the Clinical Dementia Rating (CDR) and the Mini-Mental State Examination (MMSE). The severity of PD was assessed using the Modified Hoehn and Yahr scale. Finally, a peripheral-blood sample was collected to analyze the gene expression of APOE. 

The inclusion criteria were adult patients (over 20 years old) willing to cooperate and join the study. All included patients were diagnosed with ARHL, dementia, or PD. We excluded patients of pregnancy, breast-feeding, and/or other mental illness that may interfere with the completion of the examinations. 

Informed consents were obtained. This study was conducted according to the guidelines laid down in the Declaration of Helsinki, and all procedures involving research study participants were approved by the institutional review board of Chang Gung Medical Foundation (reference number: 202002487B0). 

### 2.2. Assessment Tools

#### 2.2.1. Analyzing APOE Genotype from Peripheral-Blood Samples

Blood samples were collected to establish genotyping for the single-nucleotide polymorphisms (SNPs) rs7412 and rs429358 in APOE (gene map *locus 19q13.2*) using the PCR SNP genotyping system. Detailed instructions can be found in the user manual of the QIAamp DNA Blood Mini Kit (QIAgen, Hilden, Germany).

To determine the APOE genotype, we used the TaqMan Assay Real-Time PCR and allele-discrimination assays (commercialized by Thermo Fisher Scientific, Waltham, MA, USA). TaqMan probes were used for the SNPs *rs429358* (*C_3084793_20, 388T>C*) and *rs7412* (C_904973_10, 526C>T) within APOE exon 4 to detect nucleotide variant T or C. Based on the *rs429358/rs7412* combination within the same APOE gene, we determined the following genotypes: ε2 (T/T), ε3 (T/C), and ε4 (C/C). There were six possible allelic combinations: ε2/ε2, ε2/ε3, ε2/ε4, ε3/ε3, ε3/ε4, and ε4/ε4. 

#### 2.2.2. Hearing Assessment

PTA was used. Cautions were paid to dementia or PD patients who might have difficulty conducting behavioral hearing examinations due to poor cooperation and intelligence. Because of the out-patient setting, all our participants were able to carry out a complete hearing exam. The hearing threshold was calculated by using the mean threshold at 500 Hz, 1000 Hz, 2000 Hz, and 4000 Hz. According to the 1947 American Medical Association method, the binaural hearing handicap (%) = [(Better ear threshold − 25) × 1.5 × 5 + (worse ear threshold − 25) × 1.5]/6. 

#### 2.2.3. Cognitive Function Assessment

The assessment tools were conducted under the guidance of professionals in our out-patient settings, including the CDR Dementia Staging Instrument (scale from 0 = no dementia to 3 = severe cognitive impairment) [15] and the MMSE (measures orientation, attention, memory, language, and visual–spatial skills, scored from 0 to 30; a lower score represents greater cognitive impairment) dementia questionnaires [16].

#### 2.2.4. Staging of Parkinson’s Disease–Modified Hoehn and Yahr Scale

The Modified Hoehn and Yahr stage is a disease-stage rating scale that describes motor deficits in patients with PD (range 1–5, with higher scores indicating more severe motor deficits) [17]. The scale focuses on unilateral vs. bilateral disease and postural reflex impairment and can be used regardless of whether patients are receiving dopaminergic therapy. Severe symptoms are defined with a Modified Hoehn and Yahr scale greater than 2. In this study, the scale was determined by the neurologist Dr. Lin, one of the co-authors, who has more than twenty years of primary-care experience in PD. 

### 2.3. Statistical Analysis

All statistical analyses were performed using the SPSS software (PASW Statistics for Windows, version 21.0; IBM Corp., Armonk, NY, USA). For continuous variables, the data were expressed as mean ± standard deviation. The demographic data of the patients were compared between those with unimpaired hearing and impaired hearing by Student’s (pair) *t*-test. A *p*-value < 0.05 was considered statistically significant. 

## 3. Results

### 3.1. Demographic and Clinical Characteristics of Study Population

From 2022 to 2023, a total of 72 out-patients diagnosed with hearing loss or PD were enrolled in this study prospectively. There were 44 males and 28 females, with an average age of 64.4 ± 9.1 years (range 45–90 years old). Twenty-three participants had unimpaired hearing (≤25 decibels in both ears); 49 had hearing impairment. Thirty-one participants had non-dementia scores on both the CDR and MMSE dementia questionnaires, while the remaining 41 were classified as the dementia group (Table 1). Further classification of the types of dementia or cognitive impairment was not performed. The demographic data and hearing threshold are listed in Table 1. The dementia group had older age (71.0 ± 7.4 vs. 62.5 ± 8.9 years, *p* < 0.001) and a worse hearing threshold in both ears (47.1 ± 24.4 vs. 31.7 ± 22.1 dB, *p* = 0.006).

### 3.2. APOE Gene Expression and Dementia

All cases received blood tests for the APOE gene expression. The results showed that there were one ε2/ε2, five ε2/ε3, one ε2/ε4, fifty-six ε3/ε3, eight ε3/ε4, and one ε4/ε4. Ten patients carried the APOE ε4 allele, with a higher prevalence of dementia compared to those who did not carry the allele (9/10 vs. 32/62, *p* = 0.036; Table 2). Patients with the APOE ε4 allele seemed to have more hearing loss, but the correlations did not reach statistical significance. There was no association between PD and the APOE ε4 allele found in this study. 

### 3.3. APOE Gene Expression and Parkinson’s Disease Severity

There were 58 patients diagnosed with PD, with 14 of them classified as having severe symptoms (Modified Hoehn and Yahr scale > 2) and 44 having mild PD symptoms (Modified Hoehn and Yahr scale ≤ 2; Table 3). Patients with severe PD were found to have older average age (72.3 ± 7.4 vs. 64.5 ± 8.7 years, *p* = 0.003), a worse hearing threshold in both ears (49.6 ± 28.3 vs. 30.3 ± 17.8 dB, *p* = 0.028), and more dementia cases (12/14 vs. 18/44, *p* = 0.006). However, severe PD patients did not show a significantly higher prevalence of the APOE ε4 allele. 

## 4. Discussion

Hearing loss is an important risk factor for dementia, and it should be well managed by usage of hearing aids and protection of ears from excessive noise exposure [18]. According to the Lancet report, there are 12 risk factors that should be modified to prevent or delay up to 40% of dementias, such as hypertension, diabetes, smoking, air pollution, alcohol, and hearing loss. Among them, hearing impairment accounted for up to 9% of the relative contribution to dementia. During that report, an increased risk of dementia was found (OR 1.3, 95% CI 1.0–1.6) per 10 dB of worsening of hearing loss.

In the population-based cohort study, collected data from the National Health Insurance Research Database of Taiwan showed that hearing loss was associated with increased risk of dementia, especially in patients aged 45 to 64 years [19]. A person with mild hearing loss has a two-fold risk of having dementia, moderate has three-fold, and severe has a five-fold risk [3]. Our research also has similar findings. Although the etiology still remains unclear, hearing-impaired adults were associated with steeper temporal-lobe volume loss, including in the hippocampus and entorhinal cortex [18]. 

The prevalence of dementia, PD, and hearing loss all increases along with age. Our inclusion criteria were patients over 20 years old. Actually, it is uncommon that PD is diagnosed in people younger than 50. PD patients younger than 40 are referred to as having young-onset Parkinson’s, accounting for only two percent of all PD cases. The age of patients in our cohort ranged from 45 to 90 years old, so the large heterogeneity of age is a limitation in this study. Due to the limited case number, we did not perform further stratification of different age groups. In the future, we should focus on the older-aged human populations, probably at 60–80 years old, as our treatment target. 

The pathogenesis of these neurodegenerative diseases may have complex interactions of multifactor associations with ageing and genetic disorders. In this study, we found that hearing loss is more common in the dementia and severe PD patients. Patients carrying the APOE ε4 allele had a higher prevalence of dementia [13]. To our knowledge, this is the first study on the comparison of the three disease entities based on the gene expression of APOE.

Whether hearing impairment is involved in the intrinsic disease progression of PD or is just secondary to a more complex aging process remains to be determined. There is growing evidence from epidemiology and database studies that shows PD patients have a worse hearing threshold than the population of the same age [20,21]. PD seemed to accelerate ARHL, especially in high-frequency loss [21,22]. Upon diagnosis of PD, the hearing threshold would keep worsening over time. Hearing loss correlates with an increased risk of PD, with an adjusted hazard ratio (HR) of PD of 1.53 for the hearing-loss patients [5]. Untreated hearing loss would worsen the cognitive dysfunction in PD patients. Our findings show that hearing loss is more common in severe PD than in mild PD patients.

The relationship between the severity of PD and hearing loss could be reinforced by our findings, as shown in Table 3. We believed that the severity of PD had a negative impact on the hearing threshold. However, we lacked the control group to compare the severity of PD to the population without PD of the same age. Age is the key factor in the disease severity of PD and dementia, but we could not randomly control the age during patient recruitment due to the high heterogeneity. Better study design and larger case number are needed to estimate the relative risks for the PD patients in the hearing impairment by multivariable analysis. 

An important result of the present study is the lack of higher prevalence of the APOE ε4 allele in the severe PD patients compared with the mild PD group. The pathogenesis of hearing loss in PD patients may not only have pure cochlear involvement but also be caused by impaired central auditory function in the brain cortex, associated with cognitive deficits. Reduced amplitudes and prolonged latencies of vestibular evoked myogenic potential were found in PD patients with hearing loss [23]. PD patients usually encounter difficulty in understanding speech among noise, confirmed by testing the central auditory processing function [21]. The severity of PD is classified into seven stages, according to the Modified Hoehn and Yahr scale. Younger-age patients tend to have slighter symptoms and earlier stages of PD. We also found that those with severe PD have poorer hearing and language abilities.

Other research studied on the pathogenesis of PD tried to explain the occurrence of hearing loss, such as impaired cochlear transduction due to accumulation of α-synuclein [22,24]. Pisani V et al. proposed that it is a kind of dopamine-dependent cochlear dysfunction, as the otoacoustic emission amplitudes improved with levodopa substitution in PD patients [25]. A decrease in basal-ganglia-dopamine-transporter availability was found in PD patients, which results in a decline of the peripheral-hearing function. Dopamine not only is involved in the pathogenesis of PD but also plays a role in auditory processing [26]. 

In a meta-analysis of the relationship between the APOE gene and PD dementia, the dementia risk of those with the APOE ε4 allele was 1.72 times greater than that of those without ε4 [27]. Another study confirmed that the APOE ε4 allele is a major risk factor for the development of Parkinson’s disease dementia (hazard ratio = 2.41) [28]. Although the APOE ε4 allele did not link directly to PD severity in our study, it was more prevalent in dementia patients.

Our dementia group had worse PTA and SRT in both ears. There was not a causal relationship between hearing loss and cognitive decline but a mutual interaction between them. Hearing loss could have an impact on cognition, either directly via changes in auditory input of brain structures or indirectly via increased social isolation, depression, or reduced physical activity [14,19]. Reversely, the elderly might have difficulty in communicating and hearing due to cognitive decline. Hearing loss and dementia may be exaggerated due to a common cause, such as oxidative stress, smoking, or alcohol consumption. To compensate for decreased auditory input, the patient needs increased listening effort and cognitive load, which could lead to depletion of cognitive reserve [19,21]. PD patients may also progress to dementia and cognitive decline. In Table 3, a higher prevalence of dementia and worse hearing was found in the severe PD group. Both PD dementia and Alzheimer’s disease may progress to cognitive dysfunction and have similar mutual interactions with hearing loss.

Patients carrying APOE ε4 had a higher prevalence of dementia and hearing loss, which is in keeping with numerous reports in the last three decades. Though, the association between the APOE ε4 allele and hearing loss has not been well established yet. O’Grady et al. found that the APOE ε4 allele was found less in patients with sensorineural hearing loss, but no relationship was found between the ε4 allele and severity of hearing loss or severity of impairment of word recognition [29]. Kurniawan et al. found that the APOE ε4/ε4 genotype had the most severe hearing loss, and the APOE ε4 allele was associated with a two-fold increased risk of hearing impairment compared to those without the APOE ε4 allele [30]. In the animal model, apolipoprotein E knockout (ApoE KO) male mice have activated ROS in the spiral ganglion neurons and increased apoptosis rate, resulting in atherosclerotic change and ARHL [31]. Our study showed that the APOE ε4 allele has a positive correlation with ARHL, but it did not reach statistical significance. Future studies associating the APOE ε4 allele with hearing loss need to be large-population-based and longitudinal to help us know how the APOE gene impacts hearing change along with time.

Previous studies have shown that the APOE ε4 allele can be regarded as a genetic risk factor for Alzheimer’s disease and dementia [13,14]. The APOE ε4 allele is associated with increased cerebral amyloid-β pathology, but its effect on dementia is mediated by the severity of AD pathology [14,32]. It directly influences the development of α-synuclein pathology in dementia with Lewy bodies and Parkinson’s disease dementia in mouse studies [33]. Animals that expressed the APOE ε4 allele developed the most severe α-synuclein pathology and died soonest [34]. APOE ε2 may have a protective effect in addition to APOE ε4 being harmful. Expression of APOE ε4 was associated with more extensive α-synuclein pathology, neurodegeneration, and motor and memory dysfunction [35]. Because α-synuclein is located predominately in the efferent neuronal system within the inner ear, it could affect susceptibility to age-related or noise-induced hearing loss [22,24]. It is reasonable that the natural aging process combines neurodegenerative changes intrinsic to AD and interferes with cochlear transduction. APOE is a potential therapeutic target in neurodegenerative disease. 

Not all people carrying the APOE ε4 allele develop AD or dementia, whereas some people without the APOE ε4 allele do [36]. The effect of APOE ε4 on dementia risk can be modified by other factors, including genetic and environmental factors. This might imply the existence of social and environmental factors could trigger the harmful effects of this genetic risk factor. 

On the other hand, hearing protection, screening, and treatment might be used to mitigate the hearing-loss risk factor for dementia and PD. High education may buffer the effect of APOE ε4 on the clinical manifestation of dementia by enhancing cognitive reserve [32]. Utilization of hearing aids is the most common strategy in hearing rehabilitation to address hearing loss. Before using hearing aids, people need to consider communication needs, psychological well-being, individual preferences, and financial issues. Education and economy level act as confounding factors in the development of dementia, as hearing aid use is more commonly found in patients with higher education [37]. We would like to emphasize the importance of hearing intervention, though prospective studies are needed for stronger evidence whether hearing aids could improve cognitive function in PD or dementia patients. We should give early intervention to treat hearing impairment in both dementia and PD patients [38] because the severity of hearing loss was the most important factor associated with the benefit of hearing aid treatment. It is less beneficial for older adults to wear hearing aids due to challenges of cognitive decline or physical limitations [39]. 

To our knowledge, there is currently no evidence yet that hearing intervention by hearing aid or cochlear implant could improve quality of life or cognitive function of PD patients. There is no direct or indirect causal impact of hearing loss on cognition, so interventions to address hearing loss do not necessarily have a beneficial effect on cognitive function. Tests like the MMSE are not sensitive enough to subtle cognitive changes, so other measurements of the outcomes, such as spoken tests of cognition or long-term memory tests, should be applied when doing such studies in the future [38]. But, lots of studies pointed out that human interaction does help in regaining cognitive function, slowing down the aging process, and postponing dementia onset [14]. Auditory instructions are frequently used during training protocols to improve gait of PD cases, and auditory stimuli are used to treat Parkinson’s-related gait disorders and postural instability. More efforts should be conducted on the aural rehabilitation program, including sound amplification, assistive listening devices, and communication optimization strategies [21]. 

This study is limited to a relatively small number of cases found to carry APOE ε4. We enlisted patients from neurology and otolaryngology departments so that the heterogeneity of our participants would be large. The included patients could not be well randomized, leading to selection bias. A control group was lacking, so the analysis of severity of PD could not be adjusted for by age, as age would be the key factor of disease severity. Regression and multivariable analysis were not performed to show the odds ratio. Classification of the types of dementia and cognitive impairment was not performed. We did not enroll severe dementia cases due to out-patient settings. In the future, we hope to enroll more dementia cases to figure out the role of the APOE ε4 allele in hearing loss and PD.

## 5. Conclusions

Hearing loss is common in severe PD and in dementia patients. Patients carrying the APOE ε4 allele had a higher prevalence of dementia. Severe PD has a negative impact on the hearing threshold and cognitive dysfunction. Further prospective studies are needed for discerning the role of the APOE ε4 allele. 

## Figures and Tables

**Table 1:** Comparisons of hearing in dementia patients.

	Non-Dementia Group *n* = 31	Dementia Group *n* = 41	*p*-Value
Age (years)	62.5 ± 8.9	71.0 ± 7.4	<0.001 **
Gender			1.000
Male	19	25	
Woman	12	16	
Right-hearing threshold (dB)	31.9 ± 24.6	45.1 ± 24.5	0.025 *
Left-hearing threshold (dB)	31.6 ± 20.5	49.0 ± 25.9	0.002 **
Average hearing threshold (dB)	31.7 ± 22.1	47.1 ± 24.4	0.006 **
Binaural hearing impairment (%)	13.7 ± 25.2%	30.0 ± 30.8%	0.014 *
Speech reception threshold (dB)	27.9 ± 21.7	42.9 ± 27.4	0.011 *
Speech discrimination ability	91.4 ± 12.2%	82.7 ± 24.7%	0.075
Hearing			0.002 **
Unimpaired (threshold ≤ 25 dB)	16	7	
Impaired (>25 dB)	15	34	
Parkinson’s disease			0.132
No	3	11	
Yes	28	30	

* *p* < 0.05 (significant), ** *p* < 0.01 (highly significant).

**Table 2:** Comparison between patients with and without APOE ε4 allele.

	APOE ε4 Allele *n* = 10	No APOE ε4 Allele *n* = 62	*p*-Value
Age (years)	71.1 ± 5.8	66.7 ± 9.4	0.156
Gender			1.000
Male	6	38	
Woman	4	24	
Right-hearing threshold (dB)	48.6 ± 24.3	38.4 ± 25.6	0.240
Left-hearing threshold (dB)	51.1 ± 21.2	40.8 ± 25.8	0.232
Average hearing threshold (dB)	49.9 ± 22.5	39.6 ± 24.9	0.223
Binaural hearing impairment (%)	34.8 ± 30.0%	21.9 ± 29.6%	0.206
Speech reception threshold (dB)	47.0 ± 22.8	35.3 ± 26.7	0.196
Speech discrimination ability	85.2 ± 18.9	86.1 ± 21.4	0.898
Hearing			0.161
Unimpaired (threshold ≤ 25 dB)	1	21	
Impaired (>25 dB)	9	41	
Dementia			0.036 *
No	1	30	
Yes	9	32	
Parkinson’s disease			0.095
No	4	10	
Yes	6	52	

* *p* < 0.05 (significant).

**Table 3:** Comparison of severity of Parkinson’s disease (PD).

	Mild PD *n* = 44	Severe PD *n* = 14	*p*-Value
Age (years)	64.5 ± 8.7	72.3 ± 7.4	0.003 **
Gender			0.305
Male	29	7	
Woman	15	7	
Right-hearing threshold (dB)	28.6 ± 16.8	46.5 ± 28.5	0.041 *
Left-hearing threshold (dB)	31.8 ± 20.2	52.9 ± 29.3	0.024 *
Average hearing threshold (dB)	30.3 ± 17.8	49.6 ± 28.3	0.028 *
Binaural hearing impairment (%)	11.2 ± 20.3%	29.9 ± 34.9%	0.076
Speech reception threshold (dB)	25.7 ± 14.7	45.2 ± 28.8	0.029 *
Speech discrimination ability	93.0 ± 9.4%	80.7 ± 27.0%	0.118
Hearing			0.006 **
Unimpaired (threshold ≤ 25 dB)	21	1	
Impaired (>25 dB)	23	13	
Dementia			0.006 **
No	26	2	
Yes	18	12	
APOE ε4 allele	5	1	1.000
No APOE ε4 allele	39	13	

* *p* < 0.05 (significant), ** *p* < 0.01 (highly significant).

## Data Availability

The raw data supporting the conclusions of this article will be made available by the authors on request.

## References

[1] World Health Organization, WHO (2020). Deafness and Hearing Loss. https://www.who.int/news-room/fact-sheets/detail/deafness-and-hearing-loss.

[2] Kochkin S., Rogin C. (2000). Quantifying the obvious: The impact of hearing instruments on quality of life. Hear. Rev..

[3] Lin F.R., Albert M. (2014). Hearing loss and dementia—Who is listening?. Aging Ment. Health.

[4] Lin F.R., Metter E.J., O’Brien R.J., Resnick S.M., Zonderman A.B., Ferrucci L. (2011). Hearing loss and incident dementia. Arch. Neurol..

[5] Lai S.W., Liao K.F., Lin C.L., Lin C.C., Sung F.C. (2014). Hearing loss may be a non-motor feature of Parkinson’s disease in older people in Taiwan. Eur. J. Neurol..

[6] Großmann W. (2023). Listening with an Ageing Brain—A Cognitive Challenge. Laryngorhinootologie.

[7] Levine C.B., Fahrbach K.R., Siderowf A.D., Estok R.P., Ludensky V.M., Ross S.D. (2003). Diagnosis and treatment of Parkinson’s disease: A systematic review of the literature. Evid. Rep./Technol. Assess. (Summ.).

[8] Betarbet R., Canet-Aviles R.M., Sherer T.B., Mastroberardino P.G., McLendon C., Kim J.H., Lund S., Na H.M., Taylor G., Bence N.F. (2006). Intersecting pathways to neurodegeneration in Parkinson’s disease: Effects of the pesticide rotenone on DJ-1, alpha-synuclein, and the ubiquitin-proteasome system. Neurobiol. Dis..

[9] Uhlmann R.F., Larson E.B., Rees T.S., Koepsell T.D., Duckert L.G. (1989). Relationship of hearing impairment to dementia and cognitive dysfunction in older adults. JAMA.

[10] Kalluri S., Humes L.E. (2012). Hearing technology and cognition. Am. J. Audiol..

[11] Paternoster L., Martinez-Gonzalez N.A., Charleton R., Chung M., Lewis S., Sudlow C.L. (2010). Genetic effects on carotid intima-media thickness: Systematic assessment and meta-analyses of candidate gene polymorphisms studied in more than 5000 subjects. Circ. Cardiovasc. Genet..

[12] McKay G.J., Patterson C.C., Chakravarthy U., Dasari S., Klaver C.C., Vingerling J.R., Ho L., de Jong P.T., Fletcher A.E., Young I.S. (2011). Evidence of association of APOE with age-related macular degeneration: A pooled analysis of 15 studies. Hum. Mutat..

[13] Rasmussen K.L., Tybjaerg-Hansen A., Nordestgaard B.G., Frikke-Schmidt R. (2020). APOE and dementia—Resequencing and genotyping in 105,597 individuals. Alzheimer’s Dement..

[14] Wu J., Hasselgren C., Zettergren A., Zetterberg H., Blennow K., Skoog I., Hallerod B. (2020). The impact of social networks and APOE epsilon4 on dementia among older adults: Tests of possible interactions. Aging Ment. Health.

[15] Morris J.C. (1997). Clinical dementia rating: A reliable and valid diagnostic and staging measure for dementia of the Alzheimer type. Int. Psychogeriatr..

[16] Mitchell A.J. (2009). A meta-analysis of the accuracy of the mini-mental state examination in the detection of dementia and mild cognitive impairment. J. Psychiatr. Res..

[17] Goetz C.G., Poewe W., Rascol O., Sampaio C., Stebbins G.T., Counsell C., Giladi N., Holloway R.G., Moore C.G., Wenning G.K. (2004). Movement Disorder Society Task Force report on the Hoehn and Yahr staging scale: Status and recommendations. Mov. Disord..

[18] Livingston G., Huntley J., Sommerlad A., Ames D., Ballard C., Banerjee S., Brayne C., Burns A., Cohen-Mansfield J., Cooper C. (2020). Dementia prevention, intervention, and care: 2020 report of the Lancet Commission. Lancet.

[19] Liu C.M., Lee C.T. (2019). Association of Hearing Loss with Dementia. JAMA Netw. Open.

[20] Shetty K., Krishnan S., Thulaseedharan J.V., Mohan M., Kishore A. (2019). Asymptomatic Hearing Impairment Frequently Occurs in Early-Onset Parkinson’s Disease. J. Mov. Disord..

[21] Folmer R.L., Vachhani J.J., Theodoroff S.M., Ellinger R., Riggins A. (2017). Auditory Processing Abilities of Parkinson’s Disease Patients. BioMed Res. Int..

[22] Vitale C., Marcelli V., Allocca R., Santangelo G., Riccardi P., Erro R., Amboni M., Pellecchia M.T., Cozzolino A., Longo K. (2012). Hearing impairment in Parkinson’s disease: Expanding the nonmotor phenotype. Mov. Disord..

[23] Shalash A.S., Hassan D.M., Elrassas H.H., Salama M.M., Méndez-Hernández E., Salas-Pacheco J.M., Arias-Carrión O. (2017). Auditory- and Vestibular-Evoked Potentials Correlate with Motor and Non-Motor Features of Parkinson’s Disease. Front. Neurol..

[24] Akil O., Weber C.M., Park S.N., Ninkina N., Buchman V., Lustig L.R. (2008). Localization of synucleins in the mammalian cochlea. J. Assoc. Res. Otolaryngol..

[25] Pisani V., Sisto R., Moleti A., Di Mauro R., Pisani A., Brusa L., Altavista M.C., Stanzione P., Di Girolamo S. (2015). An investigation of hearing impairment in de-novo Parkinson’s disease patients: A preliminary study. Park. Relat. Disord..

[26] Garasto E., Stefani A., Pierantozzi M., Cerroni R., Conti M., Maranesi S., Mercuri N.B., Chiaravalloti A., Schillaci O., Viziano A. (2023). Association between hearing sensitivity and dopamine transporter availability in Parkinson’s disease. Brain Commun..

[27] Pang S., Li J., Zhang Y., Chen J. (2018). Meta-Analysis of the Relationship between the APOE Gene and the Onset of Parkinson’s Disease Dementia. Park. Dis..

[28] Real R., Martinez-Carrasco A., Reynolds R.H., Lawton M.A., Tan M.M.X., Shoai M., Corvol J.C., Ryten M., Bresner C., Hubbard L. (2023). Association between the LRP1B and APOE loci and the development of Parkinson’s disease dementia. Brain.

[29] O’Grady G., Boyles A.L., Speer M., DeRuyter F., Strittmatter W., Worley G. (2007). Apolipoprotein E alleles and sensorineural hearing loss. Int. J. Audiol..

[30] Kurniawan C., Westendorp R.G., de Craen A.J., Gussekloo J., de Laat J., van Exel E. (2012). Gene dose of apolipoprotein E and age-related hearing loss. Neurobiol. Aging.

[31] Kim Y.Y., Chao J.R., Kim C., Kim B., Thi-Thanh Nguyen P., Jung H., Chang J., Lee J.H., Suh J.G. (2020). Hearing loss through apoptosis of the spiral ganglion neurons in apolipoprotein E knockout mice fed with a western diet. Biochem. Biophys. Res. Commun..

[32] Wang H.X., Gustafson D.R., Kivipelto M., Pedersen N.L., Skoog I., Windblad B., Fratiglioni L. (2012). Education halves the risk of dementia due to apolipoprotein ε4 allele: A collaborative study from the Swedish brain power initiative. Neurobiol. Aging.

[33] Fyfe I. (2020). APOE(*)ε4 promotes synucleinopathy. Nat. Rev. Neurol..

[34] Davis A.A., Inman C.E., Wargel Z.M., Dube U., Freeberg B.M., Galluppi A., Haines J.N., Dhavale D.D., Miller R., Choudhury F.A. (2020). APOE genotype regulates pathology and disease progression in synucleinopathy. Sci. Transl. Med..

[35] Zhao N., Attrebi O.N., Ren Y., Qiao W., Sonustun B., Martens Y.A., Meneses A.D., Li F., Shue F., Zheng J. (2020). APOE4 exacerbates α-synuclein pathology and related toxicity independent of amyloid. Sci. Transl. Med..

[36] Rizzuto D., Fratiglioni L. (2014). Lifestyle factors related to mortality and survival: A mini-review. Gerontology.

[37] Engdahl B., Aarhus L. (2024). Prevalence and predictors of self-reported hearing aid use and benefit in Norway: The HUNT study. BMC Public Health.

[38] Dawes P., Völter C. (2023). Do hearing loss interventions prevent dementia?. Z. Gerontol. Geriatr..

[39] Naylor G., Dillard L., Orrell M., Stephan B.C.M., Zobay O., Saunders G.H. (2022). Dementia and hearing-aid use: A two-way street. Age Ageing.

